# Appetite sensation improvement by synbiotic supplementation in patients with metabolic syndrome: A randomized controlled clinical trial

**DOI:** 10.1002/fsn3.4124

**Published:** 2024-04-08

**Authors:** Fatemeh Hosseini, Mahboube Pourjam, Soheila Mirzaeian, Mozhgan Karimifar, Awat Feizi, Mohammad Hassan Entezari, Sahar Saraf‐Bank

**Affiliations:** ^1^ Department of Clinical Nutrition, School of Nutrition and Food Science, Nutrition and Food Security Research Center Isfahan University of Medical Sciences Isfahan Iran; ^2^ St Boniface Hospital University of Manitoba Winnipeg Manitoba Canada; ^3^ Isfahan Endocrine and Metabolism Research Center Isfahan University of Medical Sciences Isfahan Iran; ^4^ Department of Biostatistics and Epidemiology Isfahan University of Medical Sciences Isfahan Iran; ^5^ Department of Community Nutrition, School of Nutrition and Food Science, Nutrition and Food Security Research Center Isfahan University of Medical Sciences Isfahan Iran; ^6^ Supportive and Palliative Care Department Isfahan University of Medical Sciences Isfahan Iran

**Keywords:** inflammation, insulin, metabolic syndrome, probiotics, satiety, synbiotic

## Abstract

The potential link between dysbiosis, features of metabolic syndrome (MetS), inflammation, and sensation impairment has been recently recognized. However, in this context, there are few indications available regarding the effects of co‐supplementation with *Bacillus indicus*, *Bacillus coagulans*, and fructooligosaccharide (FOS) prebiotics on patients with MetS. Therefore, this study aimed to investigate the effects of synbiotic supplementation on glycemic indices, inflammatory biomarkers, and appetite among adults with MetS. This study is a randomized, double‐blind, placebo‐controlled clinical trial conducted in the Endocrine and Metabolism Research Center outpatient clinic in Isfahan, Iran. Fifty‐eight MetS patients were randomly assigned to receive either synbiotics (*n* = 29) or placebo (*n* = 29) supplementation twice per day for 8 weeks. Finally, 55 patients were recruited for analyses (28 in the intervention group and 27 in the placebo group). Random permuted blocks and a computer‐generated random number table were used for treatment allocation. No adverse effects were reported during the study. There were no significant differences in glycemic indices and inflammatory markers within‐ and between groups (all *p* > .05). However, a significant increase in the sensation of fullness was documented in the synbiotic group. In conclusion, the eight‐week treatment did not improve glycemic control and inflammatory markers. Nevertheless, it demonstrated potential efficacy in enhancing participants' appetite sensations, warranting further evaluation in longer intervention periods during future clinical trials.

## Graphical abstract

We assessed the effects of synbiotic supplementation on fasting blood sugar (FBS), fasting insulin, homeostasis assessment model for insulin resistance (HOMA‐IR), quantitative insulin sensitivity check index (QUICKI), tumor necrosis factor‐α (TNF‐ α), high‐sensitivity C‐reactive protein (hs‐CRP), and appetite among metabolic syndrome (MetS) patients. Synbiotic supplementation did not improve glycemic control and inflammatory markers at the end of the study. Synbiotic supplementation may be effective on fullness sensation in subjects with MetS.

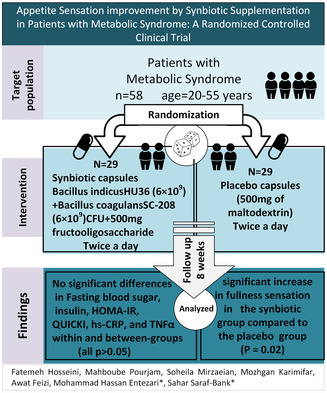

## 1 INTRODUCTION

Metabolic syndrome (MetS), a significant health issue, encompasses a cluster of risk factors, such as dyslipidemia, arterial hypertension, abdominal obesity, impaired fasting glycemia, and a proinflammatory condition (Alberti et al., 2009). These components of MetS play a crucial role in the development of type 2 diabetes and cardiovascular diseases (CVDs). Previous research indicates that individuals with MetS, compared to those without the syndrome, face a higher risk of danger of type 2 diabetes (five times higher) and CVD (two times higher) (Mottillo et al., 2010). Furthermore, there has been a notable global increase in the prevalence of MetS over the past two decades (Alberti et al., 2009; Ivezić‐Lalić et al., 2013), underscoring the urgency of addressing MetS as a public health priority (Alberti et al., 2009).

Researchers have recently taken interest in the role of the intestinal microbiota in the development of components of MetS, including obesity, altered glucose metabolism, dyslipidemia, and systemic inflammation (Cavalcanti Neto et al., 2018; Festi et al., 2014; Shen et al., 2013). Moreover, the intestinal microbiota plays a crucial role in regulating metabolic pathways that control physiological sensations of hunger and satiety (Khan et al., 2016; Mohammadi‐Sartang et al., 2018). Various therapeutic approaches have been suggested to modify the composition of the intestinal microbiota. One effective strategy involves the administration of probiotics, prebiotics, and synbiotics to modulate intestinal microbiota (Ebel et al., 2014; He & Shi, 2017; Xavier‐Santos et al., 2020).

Previous studies have indicated that specific probiotic species, prebiotics, and synbiotics could improve glycemic indices, insulin resistance, inflammatory markers, energy intake, and appetite in obese individuals, as well as in those individuals with MetS and type 2 diabetes mellitus, both in rodents and human subjects (Cicero et al., 2021; Falcinelli et al., 2018; Hajipoor et al., 2022; Liu et al., 2024; Rabiei et al., 2018; Xavier‐Santos et al., 2020). They could also influence metabolic profiles through mechanisms such as the microbial production of short‐chain fatty acids (SCFAs) (McNabney & Henagan, 2017). Some human studies have demonstrated that synbiotics have a beneficial impact on MetS components and appetite (Cicero et al., 2021; Eslamparast et al., 2014; Mohammadi‐Sartang et al., 2018; Rabiei et al., 2018). Crescenzo et al. (2017) reported that the administration of *Bacillus indicus* cells to high‐fat‐fed rats improved insulin resistance and plasma inflammatory markers.

Recently, *Bacillus* species, such as *B. indicus* and *B. coagulans*, have garnered significant interest as probiotics (Cutting, 2011; Elshaghabee et al., 2017). Past research indicates that *B. coagulans* can improve dysbiosis by increasing beneficial bacterial groups (Nyangale et al., 2015) enhance glycemic status in individuals with diabetic nephropathy (Mazruei Arani et al., 2019), and reduce the total C‐reactive protein (CRP) in subjects with rheumatoid arthritis (Mandel et al., 2010). In the same vein, Crescenzo et al. (2017) reported that the administration of *B. indicus* cells to high‐fat‐fed rats improved insulin resistance and prevented the rise in systemic inflammation markers and tumor necrosis factor‐α (TNF‐α) levels. However, there is still a considerable need to assess the potential impact of these microbial strains on the cardiovascular risk factors in adults with an unhealthy metabolic profile. Moreover, some scholars recommend the use of a combination of probiotics with a prebiotic for better outcomes (Ejtahed et al., 2011). Therefore, the present study aims to evaluate the effects of supplementation with synbiotics (*B. coagulans* and *B. indicus* as probiotics and fructooligosaccharides [FOS] as a prebiotic) on fasting glucose level, fasting insulin levels, insulin resistance, insulin sensitivity, TNF‐α, and high‐sensitivity C‐reactive protein (hs‐CRP), and appetite among MetS patients.

## 2 MATERIALS AND METHODS

### 2.1 Study population

The present study was a randomized, double‐blind, placebo‐controlled, parallel clinical trial with a 1:1 allocation ratio. The study was conducted from July 2019 to January 2020. The sample was randomly selected from individuals with MetS who were referred to the Endocrine and Metabolism Research Center outpatient clinic in Isfahan, Iran, and met our study inclusion criteria. The diagnosis of MetS was based on the National Cholesterol Education Program's criteria, as outlined in the Adult Treatment Panel III report (Grundy et al., 2005). The main inclusion criteria for selecting the participants were men and women aged between 20 and 55 years who were willing to participate in the study, with a body mass index (BMI) >25 kg/m^2^, and presenting at least three of the following features: increased waist circumference (WC) (≥102 cm in men and ≥88 cm in women), elevated triglyceride (TG) levels (≥150 mg/dL), reduced high‐density lipoprotein‐cholesterol (HDL‐C) levels (≤40 mg/dL in men and ≤50 mg/dL in women), and increased fasting blood sugar (FBS) levels (≥100 mg/dL). Although blood pressure ≥130/85 mmHg is one of the Adult Treatment Panel III criteria for metabolic syndrome, the four common MetS determinants, which are more prevalent in individuals with overweight, were considered as inclusion criteria.

Patients who were on antidiabetic medications (except Metformin, Acarbose, and Pioglitazone), lipid‐lowering drugs (except subgroups of statins, as these drugs have been found to have minimal or null effect on increasing insulin; DeMarsilis et al., 2022), anti‐inflammatory medications, antipsychotic drugs, as well as nicotinic acid and drugs affecting appetite, were excluded. Additionally, individuals who were participating in a weight loss or weight gain program, undergoing antibiotic treatment, concurrently using supplements (including vitamins, minerals, antioxidants, fiber, and pro‐, pre‐, or synbiotic supplements), and those allergic to pro‐, pre‐, or synbiotic, and maltodextrin were excluded from the research. Moreover, individuals with any of the following conditions were excluded: suspected or definite history of alcohol use, current smoking, any chronic gastrointestinal disorder, cardiovascular disease, cancer, renal, liver, lung, and thyroid disorders, eating disorders, pregnancy, lactating, and menopausal women. Additionally, patients would have been excluded from the experiment if they had missed more than 10%t of the supplement dose, changed medication, or modified their lifestyle. It is important to note that the participants included in the study were selected from the outpatient clinic of a research center and had attended several educational sessions on controlling regarding cardiovascular risk factors. Therefore, they possessed sufficient knowledge of recommendations for improving determinants of MetS. The present trial was registered in the Iranian Registry of Clinical Trials (IRCT) (http://www.IRCT.ir, IRCT20140208016529N4).

Fifty‐eight eligible patients who met the inclusion criteria signed a written informed consent form and willingly agreed to participate in the study. The investigation was conducted in accordance with the guidelines in the Declaration of Helsinki, and the study protocol was approved by the Ethics Committee of Isfahan University of Medical Sciences (Approval code: IR‐MUI.REC.1398.182).

### 2.2 Randomization and blinding

Before the research began, all eligible volunteers were assigned specific identification codes. The participants were then randomly divided into two groups, each consisting of *n* = 29, using a block randomization method (using Random Allocation Software: RAS) with a block size of 4 and random digits generated using statistical software. Subjects were stratified based on BMI (25–29.9 and ≥30). A computer‐generated random number table was used by an independent person to create an allocation sequence. The allocation sequence remained blinded from the patients and study investigators until the trial's conclusion. Synbiotic and placebo capsules and packs, supplied by Parsilact Company, Shiraz, Iran, were completely identical. The company pharmacist labeled boxes and concealed codes until the end of the research. After statistical analysis, the relevant pharmacist revealed the codes for data interpretation. Therefore, the statistician and all researchers involved in enrolling participants, collecting data, and assigning interventions were blinded to participant allocation throughout the study and the final analysis.

### 2.3 Interventions

Subjects received either synbiotic supplements as capsules (*n* = 29) or identically appearing placebo capsules (*n* = 29). Participants in both the synbiotic and placebo groups were instructed to take two capsules daily, one after breakfast and one after dinner, for 8 weeks. Additionally, they were advised to store the capsules in the refrigerator after each consumption.

Each set of two synbiotic capsules contained two viable species: *B. coagulans* SC‐208 (6 × 10^9^ colony‐forming units [CFUs]) and *B. indicus* HU36 (6 × 10^9^ CFUs) along with a prebiotic (500 mg FOS). Each of the two placebo capsules consisted of 1000 mg of maltodextrin.

The patients were instructed not to modify their dietary intake and physical activity during the trial. To ensure compliance, they recorded their dietary intake for 3 days (two working days and one weekend) and physical activity diaries at baseline and week eight of the study. Compliance with capsules' consumption, any changes in patient medication, and any adverse events were assessed through weekly phone calls. Subjects were also asked to bring the remaining synbiotic/placebo capsule packages as confirmation of compliance. Intervention compliance was defined as supplement/placebo consumption of more than 90% in each group.

### 2.4 Assessment of variables

#### 2.4.1 Dietary intake and physical activity assessment

Dietary intake was assessed using three‐day food diaries (including two weekdays and one weekend) before and after the intervention. Additionally, food records were analyzed for the average daily macro‐ and micronutrient intakes of subjects using Nutritionist IV software (First Databank Inc.), modified for Iranian foods. Physical activity records were evaluated using the metabolic equivalent of task (MET) questionnaire before and after the intervention (Ainsworth et al., 2000). Participants received face‐to‐face training on completing the questionnaires, and the recorded information was verified and finalized via a phone call by the researchers.

#### 2.4.2 Measurement of anthropometric parameters

Participants' body weight and height were measured at the start and the end of the research. Participants were weighed with light clothing and without shoes, using a digital scale to the nearest 0.1 kg. Height was assessed without shoes using a scale‐mounted stadiometer (Seca, Germany) to the nearest tenth centimeter. Additionally, BMI was also calculated by dividing weight (kg) by squared height (m^2^).

#### 2.4.3 Blood collection and biochemical measurements (primary outcomes)

Ten mL fasting blood samples were collected following an overnight hour fast at the beginning and after the 8 weeks of intervention. Blood samples were centrifuged at 3000 rpm (revolutions per minute) and immediately then stored at −80°C until further analysis. Also, FBS levels were determined using the glucose oxidase (GOD) method with commercially available kits from Pars Azmoon Co., Iran. Additionally, fasting insulin and serum TNF‐α levels were measured via enzyme‐linked immunosorbent assay (ELISA) method, utilizing kits from Monobind Inc., USA for fasting insulin, and Crystal Day Biotech Co, Ltd., Shanghai, China for serum TNF‐α. Serum concentrations of hs‐CRP were determined using the enhanced turbidimetric technique with commercially available kits from Pars Azmoon Co., Iran. The homeostasis assessment model for insulin resistance (HOMA‐IR) was calculated using the formula proposed by (Matthews et al., 1985):

$$\text{HOMA}-\mathrm{IR}=\text{Fasting blood glucose mmol}/L\times \text{fasting insulin}\;\left(\mathrm{\mu IU}/\mathrm{mL}\right)/22.5$$

The conversion factor for glucose ismg/dL=mmol/L×18

The degree of insulin sensitivity was assessed using the quantitative insulin sensitivity check index (QUICKI) equation: QUICKI = 1/[log fasting insulin (μIU/mL) + log fasting glucose (mg/dL)] (Hrebicek et al., 2002).

#### 2.4.4 Appetite assessment (secondary outcome)

The participants were directed to complete a 100 mm Visual Analogue Scale (VAS) to assess appetite sensations after a 12‐hour fasting period at baseline, and at 4 and 8 weeks during the study. The VAS was used to measure hunger, satiety, fullness, prospective food consumption (PFC), and the desire to eat sweet/salty/fatty foods (Flint et al., 2000). It was completed through face‐to‐face interviews at the start and the end of the research and through phone interviews in week 4 of the study.

### 2.5 Statistical analysis

The sample size was calculated as *N* = 29 for each group based on a study by Rabiei et al. (2018) for detecting a standardized effect size of Δ = 0.6 for fasting blood sugar as our study's main outcome by considering the type one error rate of 5%, statistical power 80%, and a dropout rate of 20%. Additionally, Statistical Package for the Social Sciences (SPSS) software (version 26; SPSS Inc., Chicago, IL) was used for analyzing the data. The normality of continuous data was assessed using the Kolmogorov–Smirnov, Skewness statistics, and Q–Q plot. All normally distributed continuous variables were reported as mean ± standard deviation (SD). Non‐normally distributed continuous data were expressed as median (minimum–maximum) after undergoing logarithmic transformation. The participants' continuous and categorical characteristics, anthropometric measures, and macro‐ and micronutrient intakes in the two groups were compared using independent samples t‐test and Chi‐squared test, respectively. Within‐group analysis assessing the effects of synbiotic supplementations on FBS, fasting insulin, HOMA‐IR, QUICKI, TNF‐α, and hs‐CRP was conducted using paired samples t‐test.

Between‐group comparisons for these variables were conducted using an analysis of covariance (ANCOVA), adjusting for baseline values. Changes in appetite, both within and between groups, were assessed using repeated‐measures analysis of variance (ANOVA). For all analyses, a *p‐*value < .05 was considered statistically significant.

## 3 RESULTS

**FIGURE 1:**
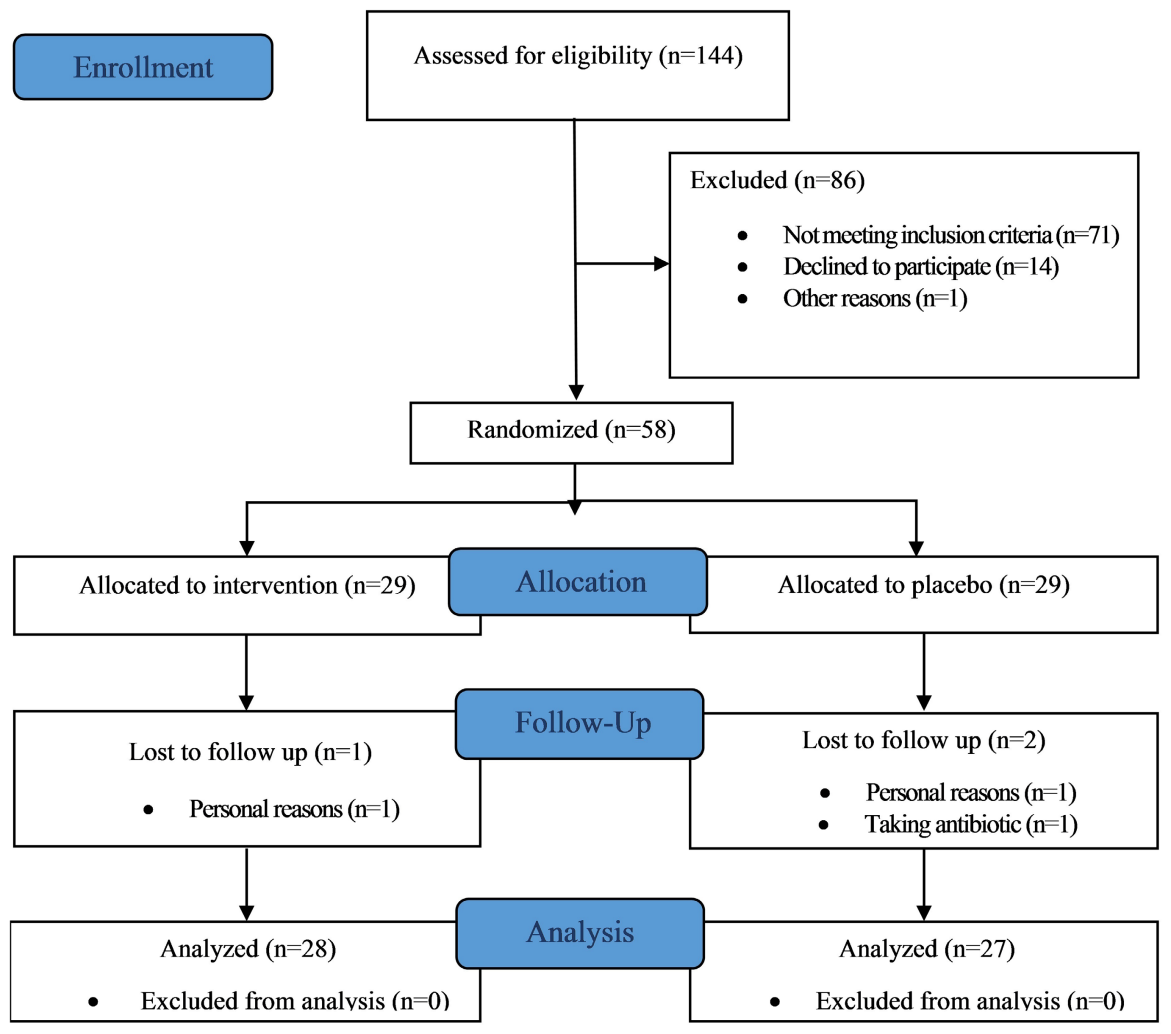
The flow diagram of recruitment of participants in the study.

The distribution of variables was found to be approximately normal, except for TNF‐α. TNF‐α variable was log‐transformed to achieve a normal distribution for subsequent analyses. Among 58 eligible individuals with MetS who enrolled in the study, three were excluded from the trial, two due to unwillingness to continue and one for taking antibiotics. Finally, the data from 55 patients who completed the intervention (28 in the synbiotic group and 27 in the placebo group) were analyzed (Figure 1). The capsule counts indicated that participants who completed the trial demonstrated satisfactory compliance with the supplementation. Both synbiotic and placebo groups reported consuming more than 90% of prescribed capsules, equivalent to more than 100 of 112 capsules. Additionally, participants did not report any adverse effects associated with the synbiotic or placebo capsules during the trial.

The baseline variables of the study participants have been presented in Table 1. No significant differences were observed in participants' baseline characteristics.

**TABLE 1:** Baseline characteristics of patients.^a^

Variable	Synbiotic group (*n* = 28)	Placebo group (*n* = 27)	*p* ^b^
Male/female	13/15	9/18	.32
Age (year)	46.89 ± 5.77	47.44 ± 7.51	.065
Weight (kg)	83.06 ± 11.75	82.98 ± 10.12	.98
BMI at the baseline (kg/m^2^)	30.92 ± 2.25	31.77 ± 3.61	.30
BMI at the end (kg/m^2^)	30.94 ± 2.17	31.78 ± 3.63	.74
WC (cm)	104 ± 3.58 (men)	105.22 ± 4.79 (men)	.50
	97.83 ± 8.93 (women)	100.72 ± 7.17 (women)	.31
FBS (mg/dL)	127.42 ± 24.74	124.25 ± 17.24	.58
TG (mg/dL)	245.07 ± 87.1	212.44 ± 63.51	.12
HDL‐C (mg/dL)	37.92 ± 7.23 (men)	39.22 ± 10.76 (men)	.73
	39.06 ± 6.52 (women)	39 ± 6.67 (women)	.97
Metformin (yes)	14 (50.0)	14 (51.9)	.89
Statins (yes)	10 (35.7)	13 (48.1)	.35

Abbreviations: BMI, body mass index; FBS, fasting blood sugar; HDL‐C, high‐density lipoprotein‐cholesterol; TG, triglyceride; WC, waist circumference.

^a^ Data are expressed as frequency, number (%), and mean ± SD.

^b^ *p‐*Values resulted from independent *t*‐tests for quantitative and chi‐square for qualitative variables between the two groups.

The three‐day food and physical activity records, compared and reported in Table 2, revealed no significant differences in the mean change of nutrient intake between the two groups.

**TABLE 2:** Dietary intakes and physical activity of study patients at the baseline and after 8 weeks of intervention.^a^

Variable	Stages	Synbiotic group (*n* = 28)	Placebo group (*n* = 27)	*p* ^b^
Total energy (kcal/day)	Before	2226.36 ± 473.31	2013.6 ± 302.31	.05
	After	2179.35 ± 426.48	1988.68 ± 333.11	.07
	Change	−47.01 ± 196.19	−24.92 ± 187.46	.67
	*p* ^c^	.21	.49	
Carbohydrate (g/day)	Before	338.30 ± 82.25	297.14 ± 50.28	.03
	After	324.47 ± 59.1	292.18 ± 55.55	.04
	Change	−13.81 ± 50.01	−4.96 ± 49.89	.51
	*p* ^c^	.15	.60	
Protein (g/day)	Before	76.53 ± 17.85	68.65 ± 19.21	.12
	After	73.27 ± 14.13	64.96 ± 15.74	.04
	Change	−3.26 ± 12.32	−3.61 ± 15.88	.91
	*p* ^c^	.17	.23	
Fat (g/day)	Before	65.92 ± 17.45	63.76 ± 17.22	.64
	After	70.61 ± 24.37	65.22 ± 17.40	.35
	Change	4.69 ± 16.18	1.46 ± 17.96	.48
	*p* ^c^	.13	.67	
Fiber (g/day)	Before	19.07 ± 7.03	15.55 ± 5.17	.04
	After	17.46 ± 4.66	15.18 ± 5.68	.11
	Change	−1.61 ± 6.73	−0.36 ± 4.72	.43
	*p* ^c^	.21	.69	
Vitamin A (μg/day)	Before	761.78 ± 402.69	557.73 ± 418.97	.10
	After	549.60 ± 383.66	579.21 ± 426.44	.78
	Change	−212.17 ± 653.08	1.57 ± 352.99	.13
	*p* ^c^	.09	.98	
Beta‐carotene (μg/day)	Before	796.77 ± 1082.13	451.15 ± 532.16	.14
	After	378.86 ± 537.28	376.33 ± 626	.98
	Change	−417.91 ± 1334.97	−74.81 ± 680.23	.23
	*p* ^c^	.1	.57	
Vitamin E (mg/day)	Before	6.63 ± 4.74	5.13 ± 2.21	.13
	After	11.71 ± 14	5.82 ± 6.20	.05
	Change	5.07 ± 13.91	0.69 ± 5.37	.13
	*p* ^c^	.06	.50	
A‐Tocopherol (mg/day)	Before	7.81 ± 3.42	6.15 ± 2.69	.05
	After	8.8 ± 12.65	5.89 ± 2.69	.24
	Change	0.98 ± 11.61	−0.26 ± 2.37	.58
	*p* ^c^	.65	.56	
Vitamin C (mg/day)	Before	109.83 ± 86.32	85.67 ± 40.96	.19
	After	86.7 ± 28.03	83 ± 43.39	.71
	Change	−23.12 ± 75.57	−2.67 ± 40.87	.21
	*p* ^c^	.11	.73	
Zinc (mg/day)	Before	9.04 ± 1.85	8.42 ± 2.21	.26
	After	8.46 ± 2.7	8.07 ± 1.64	.51
	Change	−0.58 ± 2.41	−0.35 ± 1.54	.68
	*p* ^c^	.21	.24	
Selenium (mg/day)	Before	0.15 ± 0.06	0.12 ± 0.05	.13
	After	0.15 ± 0.05	0.12 ± 0.05	.11
	Change	0.00 ± 0.06	0.00 ± 005	.95
	*p* ^c^	.97	.88	
Physical Activity (MET/h/day)	Before	26.60 ± 4.09	25.18 ± 3.77	.19
	After	26.20 ± 4.03	25.34 ± 4.08	.43
	Change	−0.39 ± 0.94	0.15 ± 1.52	.11
	*p* ^c^	.30	.59	

^a^ Data are expressed as mean ± SD.

^b^ Resulted from independent samples *t*‐test.

^c^ Resulted from paired samples *t*‐test.

The effects of synbiotic supplementation on the primary outcomes of the study are presented in Table 3. The results indicate no significant changes in glycemic and inflammatory biomarkers in within‐group analysis. Furthermore, no significant differences were observed between groups concerning these variables (all *p* > .05).

**TABLE 3:** Glycemic indices and inflammatory markers at baseline and after intervention.^a^

	Synbiotic group (*n* = 28)			*p* ^b^	Placebo group (*n* = 27)			*p* ^b^	*p* ^d^
	Baseline	End of trial	Change		Baseline	End of trial	Change		
FBS (mg/dL)	127.42 ± 24.74	126.71 ± 23.37	−0.71 ± 2.42	.77	124.25 ± 17.24	123.55 ± 16.25	−0.7 ± 2.28	.76	.82
Insulin (μIU/mL)	6.17 ± 3.26	6.08 ± 3.32	−0.08 ± 0.37	.81	8.44 ± 4.79	8.21 ± 4.07	−0.22 ± 0.97	.81	.24
HOMA‐IR	1.91 ± 1.02	1.90 ± 1.09	−0.01 ± 0.13	.92	2.5 ± 1.3	2.49 ± 1.3	−0.01 ± 0.29	.97	.33
QUICKI	0.35 ± 0.02	0.35 ± 0.02	0.001 ± 0.00	.72	0.34 ± 0.02	0.33 ± 0.02	−0.001 ± 0.00	.67	.13
hs‐CRP (mg/L)	2.8 ± 1.94	2.86 ± 1.97	0.06 ± 1.46	.81	3.06 ± 2.54	2.4 ± 1.34	−0.65 ± 2.02	.1	.1
TNF‐α^c^ (pg/mL)	32 ± 91.95	21.62 ± 47.66	−10.38 ± 46.71	.25	70.94 ± 134.37	42.56 ± 73.07	−28.37 ± 107.93	.18	.82

Abbreviations: FBS, fasting blood sugar; HOMA‐IR, homeostasis model assessment of insulin resistance; hs‐CRP, high‐sensitivity C‐reactive protein; QUICKI, quantitative insulin sensitivity check index; TNF‐α, tumor necrosis factor‐α.

^a^ Baseline and endpoint values are presented as mean ± SD, and change is presented as mean ± SE.

^b^ Resulted from paired *T*‐test.

^c^ Normalized for analysis using Ln‐transformation.

^d^ Obtained from ANCOVA, endpoint values of variables were compared that were adjusted for baseline values.

The results of synbiotic administration on secondary outcomes are presented in Table 4. Over the eight weeks of intervention, there was a significant increase in satiety and fullness sensation and a significant decline in PFC (*p*_time_: .003, <.001, and .009, respectively). However, the difference between the two groups was statistically significant only for fullness sensation (*p*_group_: .02). Moreover, a significant interaction term was observed between time and group for fullness sensation (*p*‐time × group: .002). No significant effects of synbiotic supplementation on hunger sensation or desire to eat sweet, salty, and fatty foods were observed.

**TABLE 4:** The effect of synbiotic supplementation on appetite sensations.^a^

Items	Synbiotic group (*n* = 28)			Placebo group (*n* = 27)			*p*‐Value^b^		
	Baseline	Week 4	End of trial	Baseline	Week 4	End of trial	Time	Group	Time × group
Hunger (mm)	43.71 ± 25.12	42.35 ± 22.43	41.46 ± 24.12	44.37 ± 27.42	43.33 ± 20.47	37.29 ± 21.82	.23	.87	.59
Satiety (mm)	34.10 ± 15.33	44.6 ± 15.55	49.64 ± 20.25	34.11 ± 22.17	37.03 ± 24.73	39.48 ± 25.30	.003	.20	.18
Fullness (mm)	24 ± 17.1	36.71 ± 14.99	47.46 ± 24.5	23.29 ± 20.92	27.29 ± 22.47	29.14 ± 22.98	<.001	.02	.002
PFC (mm)	52.71 ± 23.12	46.85 ± 17.86	40.5 ± 17.56	50.25 ± 25.03	50.88 ± 24.42	44.22 ± 22.12	.009	.72	.42
Desire to eat sweet (mm)	55.25 ± 34.64	65.42 ± 28.07	59.25 ± 27.3	60.48 ± 29.4	58.85 ± 28.73	61.22 ± 27.35	.59	.97	.35
Desire to eat salty (mm)	71.92 ± 27.69	76.14 ± 26.52	71.6 ± 26.82	68.59 ± 27.76	71.11 ± 24.93	76.55 ± 23.57	.30	.85	.16
Desire to eat fatty (mm)	70.57 ± 28.65	71 ± 29.91	74.64 ± 25.1	70.51 ± 33.64	72.59 ± 30.19	75.22 ± 29.41	.27	.92	.93

Abbreviation: PFC, prospective food consumption.

^a^ Data are expressed as mean ± SD.

^b^ Obtained from the two‐way (mixed) repeated‐measures ANOVA.

## 4 DISCUSSION

### 4.1 Effects of synbiotic on glycemic indices

In the present study, we aimed to examine the effects of the synbiotic supplement on FBS, insulin parameters, TNF‐α, hs‐CRP, and appetite in MetS patients. Our findings revealed that the intake of the synbiotic supplement, consisting of two *Bacillus* species with FOS, had no significant effects on FBS, insulin, HOMA‐IR, QUICKI, TNF‐α, and hs‐CRP. However, synbiotic supplementation resulted in a significant increase in fullness sensation between the groups. To the best of our knowledge, this is the first study assessing the effect of *B. coagulans* SC‐208, *B. indicus* HU36, plus FOS supplementation on glycemic and inflammatory markers, as well as appetite in MetS patients. MetS patients with a hypertriglyceridemic waist phenotype, similar to the participants in the present study, face an elevated risk of chronic diseases such as cardiovascular and diabetes (Mottillo et al., 2010; Solati et al., 2004). Therefore, conducting more research on this population is of great importance. The only study that investigated the effect of *B. indicus* in this field was conducted by Crescenzo et al. (2017), who found that eight weeks of administration of 1 × 10^10^ cells of *B. indicus*HU16 were protective against the progression of insulin resistance and high TNF‐α levels in high‐fat‐fed rats.

Until now, previous clinical trials assessing the effects of probiotic products, including yogurt (Rezazadeh et al., 2019), fermented milk (Bernini et al., 2016), and Kefir (Bellikci‐Koyu et al., 2019), as well as probiotic and synbiotic supplements (Eslamparast et al., 2014; Tenorio‐Jiménez et al., 2019) on MetS have presented contradictory results. Some trials have confirmed the beneficial effects of probiotics/synbiotics on glycemic indices (Eslamparast et al., 2014; Liang et al., 2021; Rabiei et al., 2018; Rezazadeh et al., 2019), whereas others have not found conclusive evidence of supplementation (Bernini et al., 2016; Tenorio‐Jiménez et al., 2019). In line with the results of the present study, a meta‐analysis of 10 randomized controlled clinical trials that examined the efficacy of pro/synbiotic supplementation among adults with MetS showed no significant differences in FBS, insulin, and HOMA‐IR between the intervention and placebo groups (Hadi et al., 2021). Regarding bacterial strains, two clinical trials have assessed the efficacy and safety of *B. coagulans* on diabetic nephropathy (Mazruei Arani et al., 2019) and healthy obese or hyperglycemic adults (Angelino et al., 2019). Our findings align with the results of Angelino et al. (2019), who reported that the consumption of a daily serving of whole‐grain pasta enriched with barley β‐glucans and probiotic *B. coagulans* for 12 weeks had no significant effects on plasma glucose and serum insulin. Meanwhile, Mazruei Arani et al. (2019) found that the consumption of 25 g/day of honey‐containing probiotic *B. coagulans* for 12 weeks had considerable effects on serum insulin, HOMA‐IR, and QUICKI score. These inconsistent findings could be primarily explained by the dosage of probiotics used in each study, different clinical trial designs, baseline characteristics of the study participants, the history of medication used, and the duration of the trial in different studies. In the current study, 8 weeks of treatment might not have been long enough to improve glycemic status in MetS.

Despite the absence of significant findings in our study, suggested mechanisms underlying the effect of probiotics on glycemic markers have been proposed, including modulation of the gut microbiota, alterations in intestinal environment, and regulation of immune responses (Asemi et al., 2013). Probiotics may also impact glycemic control by influencing glucose absorption in the intestines and utilizing glucose as a major energy source (Yadav et al., 2007).

### 4.2 Effects of synbiotic on inflammatory markers

The current study did not reveal any significant differences in serum hs‐CRP and TNF‐α levels within‐ and between the two groups at the end of the trial. Consistent with our results, a recent meta‐analysis of eight clinical trials investigating the effect of synbiotic supplementation in patients with MetS indicated that synbiotic supplements have a null effect on hs‐CRP levels (Arabi et al., 2022; Mazruei Arani et al., 2019). Moreover, two previous clinical trials did not find any significant effect of *B. coagulans* and *Lactobacillus reuteri* V3401 supplementation on the serum levels of TNF‐α and hs‐CRP among healthy obese and MetS patients, respectively (Angelino et al., 2019; Tenorio‐Jiménez et al., 2019). However, there is one clinical trial that demonstrated the reducing effect of *B. coagulans* on hs‐CRP (Mazruei Arani et al., 2019). However, in the current study, the lack of statistically significant effects of synbiotic supplementation on inflammatory markers might be attributed to the lower levels of these markers at the beginning of the study compared to the aforementioned study that showed significant effects of probiotic consumption on inflammation (Mazruei Arani et al., 2019; Rezazadeh et al., 2020). Furthermore, the duration of the study may not have been sufficient to improve the proinflammatory state in patients with MetS.

Despite the lack of significant changes in inflammatory markers, the potential mechanisms underlying probiotics' effects on inflammatory markers include alterations in gut microbiome diversity and the production of short‐chain fatty acids (SCFAs), such as butyrate, which interact with various cell receptors to modulate inflammation and improve metabolic disorders (Arabi et al., 2022; Canfora et al., 2015).

### 4.3 Effects of synbiotic on appetite parameters

In this study, a significant increase in fullness sensation was observed between the two groups. However, we did not observe any significant differences in other appetite parameters, including hunger, satiety, PFC, the desire to eat sweet, salty, fatty, and participants' weight between the groups after 8 weeks of intervention. Changes in appetite may not yet affect BMI, as it may take longer for this change to be reflected in physical changes. Rabiei et al. (2018) reported that synbiotic supplementation led to reduced appetite following an increase in levels of peptide YY (PYY) and glucagon‐like peptide‐1 (GLP‐1) hormones in patients with MetS. Nevertheless, a recent systematic review including 24 randomized controlled trials, involving 1587 overweight or obese participants, showed that probiotic supplementation had minimal effects on appetite hormones (Cabral et al., 2021). Overall, these contradictory results may be explained by different study designs or different strains of probiotics.

The net effect of synbiotics on appetite is still unclear, but some potential mechanisms have been suggested. One study has proposed that probiotic consumption is associated with increased production of various metabolites, including SCFAs such as propionate, acetate, and butyrate via the colonic fermentation of prebiotics. This can stimulate appetite‐related hormones (GLP‐1 and PYY), potentially via the interactions with G protein‐coupled receptors (GPCRs) on enteroendocrine cells (Cabral et al., 2021; Yadav et al., 2013). GLP‐1 is capable of inhibiting glucagon secretion, prolonging gastric emptying time, and suppressing food intake or appetite (Genta et al., 2009).

To our knowledge, this is the first study to investigate the effects of this unique combination of two species of *Bacillus* and FOS on patients with MetS. Recently, *Bacillus* species, such as *B. indicus* and *B. coagulans*, have attracted researchers' interest (Cutting, 2011). *Bacillus coagulans* produces lactic acid and exhibits characteristics of both *Lactobacillus* and *Bacillus* genera (Elshaghabee et al., 2017). Past research has shown that *B. coagulans* can improve dysbiosis by increasing beneficial bacterial groups (Nyangale et al., 2015) and glycemic status in individuals with diabetic nephropathy (Mazruei Arani et al., 2019). Furthermore, *B. indicus* is capable of producing highly stable carotenoids that are potentially related to weight control (Duc le et al., 2006; Gunanti et al., 2014; Hong et al., 2008; Neuhouser et al., 2001). However, the results of this study might be limited to the duration of the trial. It remains unclear how long supplementation needs to reach the beneficial effects of synbiotic supplementation on glycemic indices, inflammatory markers, and appetite status of MetS patients. Assessing the intestinal microbiota composition before and after probiotic supplementation is critical to evaluate the survival of probiotic bacteria and identify actual changes in participants' intestinal microbiota (Picard et al., 2005; Zhao et al., 2021). Due to budget limitations, intestinal microbiota were not evaluated in this study. Although the VAS questionnaire was used as an easy and cost‐effective tool only in the current study, it is essential to determine appetite‐related hormones for a more comprehensive understanding of the results.

The results of the present study can only be generalized to similar populations included in this study and may not apply to clinical groups or populations that have varying clinical features. Further trials with longer durations, the use of other species, and varied doses of probiotic bacteria are necessary.

## 5 CONCLUSIONS

In conclusion, the results of the present study showed that 8 weeks of synbiotic supplementation, consisting of two kinds of bacteria strains and FOS, increased fullness sensation between the two groups, while it had no significant beneficial effects on FBS, fasting insulin level, HOMA‐IR, QUICKI, TNF‐α, and hs‐CRP in patients with MetS. Future studies should be undertaken to further investigate this topic.

## AUTHOR CONTRIBUTIONS

**Fatemeh Hosseini:** Conceptualization (equal); data curation (equal); investigation (equal); methodology (equal); writing – original draft (lead); writing – review and editing (equal). **Mahboube Pourjam:** Conceptualization (equal); investigation (equal); methodology (equal); writing – review and editing (equal). **Soheila Mirzaeian:** Writing – review and editing (equal). **Mozhgan Karimifar:** Investigation (equal); writing – review and editing (equal). **Awat Feizi:** Formal analysis (lead); methodology (equal); writing – review and editing (equal). **Mohammad Hassan Entezari:** Project administration (equal); supervision (equal); writing – review and editing (equal). **Sahar Saraf‐Bank:** Project administration (equal); supervision (equal); writing – review and editing (equal).

## FUNDING INFORMATION

This project was funded by Isfahan University of Medical Sciences (No: 298015).

## CONFLICT OF INTEREST STATEMENT

The authors declare no conflict of interest.

## Data Availability

The data sets used and/or analyzed during this study are available from the corresponding author on reasonable request.
